# Clinical outcomes in high‐hypoglycaemia‐risk patients with type 2 diabetes switching to insulin glargine 300 U/mL versus a first‐generation basal insulin analogue in the United States : Results from the DELIVER High Risk real‐world study

**DOI:** 10.1002/edm2.306

**Published:** 2021-11-22

**Authors:** Sean D. Sullivan, Nick Freemantle, Rishab A. Gupta, Jasmanda Wu, Charlie J. Nicholls, Jukka Westerbacka, Timothy S. Bailey

**Affiliations:** ^1^ The CHOICE Institute School of Pharmacy University of Washington Seattle WA USA; ^2^ Comprehensive Clinical Trials Unit University College London London UK; ^3^ Accenture Florham Park NJ USA; ^4^ Sanofi US, Inc. Bridgewater NJ USA; ^5^ Sanofi Reading UK; ^6^ Sanofi Paris France; ^7^ AMCR Institute Escondido CA USA

**Keywords:** high risk, hypoglycaemia, Insulin glargine 300 units/mL, real‐world study, type 2 diabetes

## Abstract

**Aims:**

To compare 12‐month clinical effectiveness of insulin glargine 300 units/mL (Gla‐300) versus first‐generation basal insulin analogues (BIAs) (insulin glargine 100 units/mL [Gla‐100] or insulin detemir [IDet]) in patients with type 2 diabetes (T2D) who were at high risk of hypoglycaemia and switched from one BIA to a different one (Gla‐300 or Gla‐100/IDet) in a real‐world setting.

**Methods:**

DELIVER High Risk was a retrospective observational cohort study of 2550 patients with T2D who switched BIA to Gla‐300 (Gla‐300 switchers) and were propensity score‐matched (1:1) to patients who switched to Gla‐100 or IDet (Gla‐100/IDet switchers). Outcomes were change in glycated haemoglobin A1c (HbA1c), attainment of HbA1c goals (<7% and <8%), and incidence and event rates of hypoglycaemia (all‐hypoglycaemia and hypoglycaemia associated with an inpatient/emergency department [ED] contact).

**Results:**

HbA1c reductions were similar following switching to Gla‐300 or Gla‐100/IDet (−0.51% vs. −0.53%; *p* = .67), and patients showed similar attainment of HbA1c goals. Patients in both cohorts had comparable all‐hypoglycaemia incidence and event rates. However, the Gla‐300 switcher cohort had a significantly lower risk of inpatient/ED‐associated hypoglycaemia (adjusted odds ratio: 0.73, 95% confidence interval: 0.60–0.89; *p* = .002) and experienced significantly fewer inpatient/ED‐associated hypoglycaemic events (0.21 vs. 0.33 events per patient per year; *p* < .001).

**Conclusion:**

In patients with T2D at high risk of hypoglycaemia, switching to Gla‐300 or Gla‐100/IDet achieved similar HbA1c reductions and glycaemic goal attainment, but Gla‐300 switchers had a significantly lower risk of hypoglycaemia associated with an inpatient/ED contact during 12 months after switching.

## Graphical abstract

In patients with type 2 diabetes at high risk of hypoglycaemia, risk of inpatient/emergency department‐related hypoglycaemia was significantly lower during 12 months after switching to insulin glargine 300 units/mL, compared with switching to insulin glargine 100 units/mL or insulin detemir.

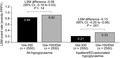

## 1 INTRODUCTION

According to the United States (US)^1^, more than 34 million Americans (10.5% of the population) had diabetes, the vast majority of whom had type 2 diabetes (T2D).^2^ Although T2D can be controlled in the early stages with metformin and lifestyle modifications, the progressive nature of the condition means that many patients will progress through successive levels of treatment intensification and will eventually require basal insulin.^3^, ^4^

In randomized controlled trials in patients with T2D, the second‐generation basal insulin analogue (BIA), insulin glargine 300 U/mL (Gla‐300), has been compared with the first‐generation BIA, insulin glargine 100 units/mL (Gla‐100), and has been shown to provide comparable glycaemic control while reducing the risk of some hypoglycaemic endpoints.^5^, ^6^, ^7^, ^8^, ^9^ These results have now also been confirmed in various meta‐analyses.^10^, ^11^, ^12^

Earlier real‐world studies of Gla‐300 have largely focused on a general diabetes population who were either initiating BIAs (DELIVER Naïve,^13^ DELIVER Naïve D,^14^ LIGHTNING^15^) or were switching from another BIA (DELIVER 1,^16^ DELIVER 2,^17^ DELIVER D,^18^ DELIVER D+,^19^ LIGHTNING^15^). While the DELIVER 3 study examined the treatment effectiveness of Gla‐300 in older patients,^20^ and DELIVER D+^19^ and LIGHTNING^15^ reported on some higher risk subgroups, several other important subgroups expected to be at increased risk of hypoglycaemia have yet to be studied in detail. Additionally, the advantages of second‐generation BIAs such as Gla‐300 for patients at high risk of hypoglycaemia in real‐world settings have not been well characterized beyond 6 months.

Several characteristics have been shown to confer an increased hypoglycaemia risk in patients with T2D, including older age,^21^ renal impairment,^22^, ^23^, ^24^ the presence of multiple comorbidities including diabetes comorbidities,^25^ and cardiovascular disease,^26^ long‐standing diabetes and/or insulin use, or the use of multiple daily injections or sulphonylureas.^27^

The objective of the DELIVER High Risk study was to compare the 12‐month clinical effectiveness of Gla‐300 versus first‐generation BIAs (Gla‐100 or insulin detemir [IDet]) in patients with T2D who were at higher risk of hypoglycaemia and were switching from one BIA to a different BIA (Gla‐100 or IDet to Gla‐300, and from Gla‐100 to IDet or from IDet to Gla‐100).

## 2 MATERIALS AND METHODS

### 2.1 Study design and data source

DELIVER High Risk was a retrospective, observational cohort study examining patients with a diagnosis of T2D treated in real‐world clinical practice settings and deemed at high risk of hypoglycaemia, who were switching from one BIA to a different BIA: from either Gla‐100 or IDet to Gla‐300 (‘Gla‐300 switchers’); or from Gla‐100 or IDet to IDet or Gla‐100 (‘Gla‐100/IDet switchers’). Only patients switching to and from these BIAs were included; patients switching from neutral protamine Hagedorn insulin were not included. Retrospective data were obtained from Accenture's Predictive Health Intelligence Environment (IBM Explorys, Cleveland, Ohio), which provides electronic medical record (EMR) data for approximately 18% of the US population. This database contains a geographically diverse spectrum of longitudinal medical data from 39 major integrated delivery networks and, importantly, represents a broad mix of patients enrolled in privately insured and government‐sponsored healthcare programmes. The database includes information on patient clinical and demographic characteristics, insurance status, healthcare encounters, diagnoses, procedure codes, and associated laboratory values and surgeries, for approximately 50 million patients.

Data were classified using common ontologies such as International Classification of Diseases, Ninth or Tenth Revisions, Clinical Modification (ICD‐9‐CM or ICD‐10‐CM) codes (for diagnoses and some procedures), Current Procedural Terminology codes (for procedures), Logical Observation Identifiers Names and Codes (for clinical and laboratory observations), and National Drug Codes (for prescriptions).

The study period was from 1 March 2014 to 30 November 2018. Patients were identified during the period 1 March 2015 to 30 November 2017 (the identification period). The date of the first prescription for Gla‐300, Gla‐100 or IDet during the identification period was defined as the index date. The 12‐month period before the index date was defined as the baseline period. Outcomes were evaluated during the 12‐month post‐index‐date follow‐up period, with no loss of patients during follow‐up.

### 2.2 Study population

Patients were included in the study if they were aged ≥18 years on the index date and had: (a) ≥1 diagnosis of T2D according to ICD‐9‐CM/ICD‐10‐CM codes^28^ (listed in Table S1); (b) ≥1 prescription of Gla‐300, Gla‐100 or IDet during the identification period; (c) ≥1 prescription of a BIA (different from the index BIA) in the 12‐month baseline period; (d) ≥12 months of baseline EMR activity (defined as any encounter with the healthcare system); (e) ≥12 months of follow‐up EMR activity; and (f) valid glycated haemoglobin A1c (HbA1c) measurements (3%–15%^1^ ^,^ ^29^) during the 6‐month baseline and 9‐ to 12‐month follow‐up. All patients also had to have ≥1 of the following seven subgroup characteristics expected to confer a high risk of hypoglycaemia. Of note, patients could have been included in more than one subgroup.

- Subgroup 1: Uncontrolled glycaemia, defined as HbA1c ≥8%.
- Subgroup 2: Switching from a combination of basal and prandial insulin.
  - At least one new prescription of prandial insulin in the 6‐month baseline before the index date or a prescription with an end date within the 6‐month baseline showing patients are still being treated with a prandial insulin during the 6‐month baseline.
- Subgroup 3: Moderate‐to‐severe renal impairment (estimated glomerular filtration rate [eGFR] <60 mL/min/1.73 m^2^, nephropathy, or end‐stage renal disease [based on ICD codes]).
- Subgroup 4: An episode of hypoglycaemia during the previous 12 weeks, moderate renal impairment (eGFR 30–59 mL/min/1.73 m^2^), long exposure (>4 years) to insulin, and/or an episode of hypoglycaemia associated with an inpatient/emergency department (ED) contact during the previous 12 months.
- Subgroup 5: Established atherosclerotic cardiovascular disease (ASCVD) (myocardial infarction, stroke, any revascularization procedure, clinically significant atherosclerosis, transient ischaemic attack, hospitalized unstable angina, amputation, congestive heart failure, 50% stenosis of any artery, symptomatic or asymptomatic coronary artery disease documented by imaging, or chronic kidney disease with eGFR ≤60 mL/min/1.73 m^2^).
- Subgroup 6: Aged ≥65 years at the index date.
- Subgroup 7: A prescription for a sulphonylurea during follow‐up.

Patients were excluded if they had type 1 diabetes according to ICD‐9‐CM/ICD‐10‐CM codes^28^ (listed in Table S1) or prescriptions of more than one BIA on the index date.

Baseline patient data extracted from the EMRs included: age, sex, race, US geographic region, insurance type, comorbidities during 12‐month baseline (identified according to ICD‐9‐CM and ICD‐10‐CM diagnosis codes detailed in Table S2), HbA1c (last value between 3% and 15% during baseline), hypoglycaemia (defined in Table S1) incidence (% of patients with ≥1 episode) and event rate (events per patient per year [PPPY]), body mass index (BMI; closest measurement to index date), oral anti‐diabetic medication use during 12‐month baseline, and concomitant non‐diabetes medication use during 12‐month baseline.

### 2.3 Propensity score‐matching

To minimize confounding, Gla‐300 switchers were propensity score‐matched^30^ (1:1) to Gla‐100/IDet switchers using a propensity score derived from baseline demographics and clinical characteristics. A greedy nearest‐neighbour algorithm was used for propensity score‐matching. This algorithm selects a subject switched to Gla‐300 and then selects a matched control subject (the subject switched to Gla‐100/IDet whose propensity score is closest to that of the selected subject). Once a match was made, patients were not reconsidered for further matching. Propensity scores were matched using two to eight decimal places (with a calliper width of 0.01), sequentially from highest to lowest digit match.

### 2.4 Outcomes

Outcomes were compared between propensity score‐matched Gla‐300 switchers and Gla‐100/IDet switchers. HbA1c outcomes were follow‐up HbA1c, captured as the last value during 9‐ to 12‐month follow‐up; HbA1c change from baseline; and the proportions of patients reaching common glycaemic goals (HbA1c <7% and HbA1c <8%) during follow‐up. Hypoglycaemia outcomes included the incidence and event rates of any severity of hypoglycaemia (‘all‐hypoglycaemia’; identified by ICD‐9‐CM/ICD‐10‐CM codes and/or plasma glucose level ≤70 mg/dL [3.9 mmol/L]), and incidence and event rates of hypoglycaemia associated with an inpatient or ED contact to 12‐month follow‐up.

Analyses were conducted on the total matched populations of Gla‐300 and Gla‐100/IDet switchers. Additionally, outcomes were analysed for each of the seven individual hypoglycaemia‐risk subgroups (see section 2.2). Matching quality for the individual subgroups was assessed, and where characteristics differed, adjusted analyses accounting for any baseline characteristics were undertaken.

### 2.5 Statistical analysis

Categorical variables are presented as frequencies and percentages, and continuous variables as means and standard deviations (SDs). Patients with missing data were classified as ‘unknown’ for any missing variables. HbA1c reduction was analysed using Student t‐tests, and HbA1c goal attainment was compared using Fisher's exact tests. Hypoglycaemia incidence was calculated using logistic regression and adjusted odds ratios (aORs), along with 95% confidence intervals (CIs) and *P*‐values, were calculated. For event rates of hypoglycaemia, least‐square mean (LSM) differences, 95% CIs, and *P*‐values were calculated using a generalized linear model.

### 2.6 Sensitivity analyses

Sensitivity analyses were performed for HbA1c outcomes by considering latest HbA1c laboratory observations during 3–6 and 6–9 months post‐index date as the follow‐up HbA1c. For the hypoglycaemia outcomes, a sensitivity analysis was performed using only hypoglycaemia events identified by ICD‐9‐CM/ICD‐10‐CM codes. All analysed subjects had at least two HbA1c measurements, with the measurements closest in time to the index date and follow‐up milestone selected.

## 3 RESULTS

### 3.1 Study population

**FIGURE 1:**
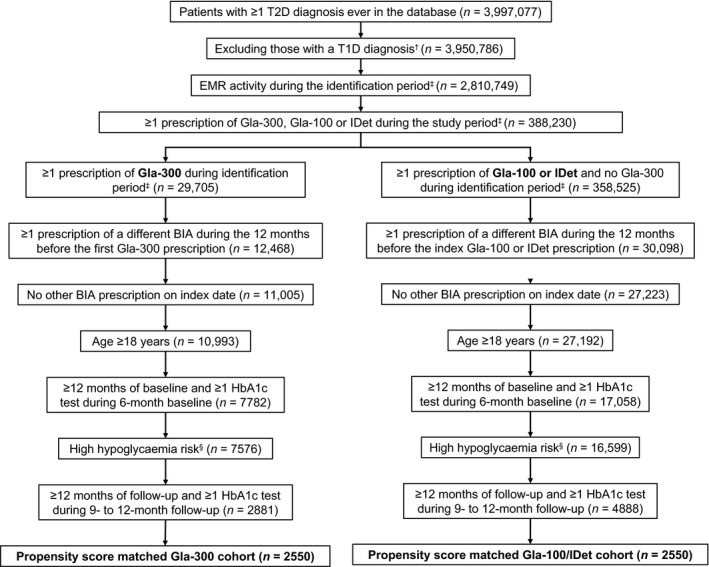
Study flow chart. ^†^Type 1 diabetes according to ICD‐9‐CM/ICD‐10‐CM codes ^28^(listed in Supporting Information Table S1). ^‡^1 March 2015 to 30 November 2017. §Patients had to have ≥1 of the seven subgroup characteristics listed in Section 2.2. Abbreviations: BIA, basal insulin analogue; EMR, electronic medical record; Gla‐100, insulin glargine 100 units/mL; Gla‐300, insulin glargine 300 units/mL; HbA1c, glycated haemoglobin A1c; ICD‐9‐CM/ICD‐10‐CM, International Classification of Diseases, Ninth or Tenth Revisions, Clinical Modification; IDet, insulin detemir; T1D, type 1 diabetes; T2D, type 2 diabetes

As shown in the study flow diagram (Figure 1), 2881 patients who switched to Gla‐300 and 4888 patients who switched to Gla‐100/IDet met the inclusion criteria. Outcomes were analysed in 2550 propensity score‐matched patients in each of the Gla‐300 switcher and Gla‐100/IDet switcher cohorts.

Before propensity score‐matching, there were some statistically significant differences in baseline characteristics between Gla‐300 switchers and Gla‐100/IDet switchers, including age, race, insurance type, HbA1c, BMI, some diabetes therapies and other concomitant medications, some comorbidities (hyperlipidaemia, obesity, neuropathy), hypoglycaemia, and healthcare utilization (Table 1). After propensity score‐matching, all baseline characteristics had a standard mean difference <0.1, indicative of a good balance in baseline characteristics between the cohorts (Table 1). There were no significant differences (with an alpha level set conservatively at *p *< .15) in any of the baseline characteristics between the matched cohorts.

**TABLE 1:** Baseline patient characteristics before and after propensity score‐matching

Characteristics	Before propensity score‐matching				After propensity score‐matching			
	Gla‐300 switchers *N* = 2881	Gla‐100/IDet switchers *N* = 4888	*p*‐value	SMD	Gla‐300 switchers *N* = 2550	Gla‐100/IDet switchers *N* = 2550	*p*‐value	SMD
Age, years, mean (SD)	60.1 (11.8)	62.4 (12.5)	<.001	0.19	60.7 (11.9)	60.5 (11.9)	.572	0.02
Male, n (%)	1393 (48.4)	2297 (47.0)	.247	0.03	1218 (47.8)	1213 (47.6)	.889	0.00
Race, n (%)								
White	1772 (61.5)	2924 (59.8)	.004	0.03	1549 (60.7)	1533 (60.1)	0.965	0.01
African American	456 (15.8)	726 (14.9)		0.03	404 (15.8)	419 (16.4)		0.02
Other (multiracial, native American or Alaskan native, Other)	104 (3.6)	160 (3.3)		0.02	90 (3.5)	80 (3.1)		0.02
Unknown	549 (19.1)	1078 (22.1)		0.07	507 (19.9)	518 (20.3)		0.01
Insurance type, n (%)								
Commercial	966 (33.5)	1350 (27.6)	<.001	0.13	807 (31.6)	837 (32.8)	.565	0.03
Medicaid	355 (12.3)	513 (10.5)		0.06	301 (11.8)	301 (11.8)		0.00
Medicare	1216 (42.2)	2288 (46.8)		0.09	1127 (44.2)	1095 (42.9)		0.03
Others (military, workers' comp, other public)	65 (2.3)	156 (3.2)		0.06	63 (2.5)	58 (2.3)		0.01
Unknown	279 (9.7)	581 (11.9)		0.07	252 (9.9)	259 (10.2)		0.01
US geographic region, n (%)								
Midwest	2115 (73.4)	3525 (72.1)	.674	0.03	1885 (73.9)	1883 (73.8)	.860	0.00
Northeast	46 (1.6)	119 (2.4)		0.06	43 (1.7)	35 (1.4)		0.03
South	582 (20.2)	1077 (22.0)		0.04	517 (20.3)	527 (20.7)		0.01
West	138 (4.8)	164 (3.4)		0.07	105 (4.1)	105 (4.1)		0.00
Unknown	0	3 (0.1)		0.00	N/A	N/A		N/A
Physician specialty associated with index event, *n* (%)								
Primary care practitioners	1132 (39.3)	1781 (36.4)	.113	0.06	983 (38.5)	968 (38.0)	.691	0.01
Endocrinologist	196 (6.8)	199 (4.1)		0.12	133 (5.2)	132 (5.2)		0.00
Internal medicine	527 (18.3)	864 (17.7)		0.02	479 (18.8)	491 (19.3)		0.01
Other HCPs/Unknown	1026 (35.6)	2044 (41.8)		0.13	955 (37.5)	959 (37.6)		0.00
Baseline HbA1c^†^, mean (SD)	9.11 (1.86)	8.83 (1.95)	<.001	0.15	9.05 (1.87)	9.09 (1.93)	.401	0.02
HbA1c^†^ category, n (%)								
<7%	273 (9.5)	738 (15.1)	.182	0.17	266 (10.4)	257 (10.1)	.868	0.01
7% to <8%	540 (18.7)	1128 (23.1)		0.11	514 (20.2)	498 (19.5)		0.02
8% to <9%	708 (24.6)	1037 (21.2)		0.08	601 (23.6)	630 (24.7)		0.03
≥9%	1360 (47.2)	1985 (40.6)		0.13	1169 (45.8)	1165 (45.7)		0.00
Baseline BMI, kg/m^2^, mean (SD)	35.4 (7.4)	34.2 (7.6)	<.001	0.15	35.16 (7.4)	35.0 (7.5)	.363	0.03
Injectable therapy during 12‐month baseline, *n* (%)								
GLP‐1 RA	530 (18.4)	516 (10.6)	<.001	0.22	371 (14.5)	380 (14.9)	.752	0.01
RAI	1637 (56.8)	2594 (53.1)	.001	0.00	1417 (55.6)	1402 (55.0)	.693	0.01
GLP‐1 RA or RAI	1887 (65.5)	2879 (58.9)	<.001	0.00	1598 (62.7)	1603 (62.9)	.908	0.00
OAD therapy during 12‐month baseline, *n* (%)								
Number of OADs, mean (SD)	1.2 (0.8)	1.1 (0.8)	<.001	0.15	1.2 (0.8)	1.2 (0.8)	.914	0.00
OADs	2071 (71.9)	3204 (65.5)	<.001	0.14	1793 (70.3)	1782 (69.9)	.760	0.01
SGLT2 inhibitor	401 (13.9)	387 (7.9)	<.001	0.19	295 (11.6)	286 (11.2)	.724	0.01
DPP‐4 inhibitor	610 (21.2)	868 (17.8)	<.001	0.09	531 (20.8)	517 (20.3)	.652	0.01
Sulphonylureas	826 (28.7)	1502 (30.7)	.058	0.05	753 (29.5)	759 (29.8)	.878	0.01
Metformin	1484 (51.5)	2338 (47.8)	.002	0.07	1299 (50.9)	1307 (51.3)	.845	0.01
Thiazolidinediones	154 (5.3)	194 (4.0)	.005	0.07	127 (5.0)	121 (4.7)	.745	0.01
Alpha‐glucosidase inhibitor	9 (0.3)	15 (0.3)	1.000	0.00	8 (0.3)	13 (0.5)	.382	0.03
Meglitinides	33 (1.1)	52 (1.1)	.736	0.01	28 (1.1)	30 (1.2)	.895	0.01
Comorbidities/diabetic complications during 12‐month baseline, *n* (%)								
Elixhauser Comorbidity Index, mean (SD)	4.1 (2.5)	4.6 (2.9)	<.001	0.17	4.2 (2.6)	4.2 (2.6)	.813	0.01
Hypertension	2464 (85.5)	4174 (85.4)	.894	0.00	2182 (85.6)	2145 (84.1)	.160	0.04
Hyperlipidaemia	2429 (84.3)	4000 (81.8)	.005	0.07	2134 (83.7)	2128 (83.5)	.850	0.01
Obesity	1223 (42.5)	1940 (39.7)	.017	0.06	1047 (41.1)	1055 (41.4)	.842	0.01
Neuropathy	913 (31.7)	1334 (27.3)	<.001	0.10	777 (30.5)	753 (29.5)	.482	0.02
Retinopathy	293 (10.2)	540 (11.0)	.239	0.03	265 (10.4)	271 (10.6)	.819	0.01
Nephropathy	259 (9.0)	429 (8.8)	.772	0.01	230 (9.0)	211 (8.3)	.370	0.03
Hypoglycaemia during 12‐month baseline								
Number of patients with hypoglycaemic events, *n* (%)	678 (23.5)	1399 (28.6)	<.001	0.12	631 (24.7)	609 (23.9)	.493	0.02
Number of hypoglycaemic events, mean (SD)	0.6 (1.5)	0.8 (2.0)	<.001	0.13	0.6 (1.6)	0.6 (1.6)	.556	0.02
Concomitant medication during 12‐month baseline, *n* (%)								
ACE inhibitors	1371 (47.6)	2308 (47.2)	.760	0.01	1199 (47.0)	1207 (47.3)	.844	0.01
Angiotensin receptor blockers	314 (10.9)	505 (10.3)	.444	0.02	268 (10.5)	263 (10.3)	.855	0.01
Calcium channel blockers	365 (12.7)	777 (15.9)	<.001	0.09	340 (13.3)	340 (13.3)	1.000	0.00
Beta‐blockers	960 (33.3)	1857 (38.0)	<.001	0.10	877 (34.4)	865 (33.9)	.745	0.01
Statins	2070 (71.9)	3565 (72.9)	.305	0.02	1852 (72.6)	1848 (72.5)	.925	0.00
Diuretics	170 (5.9)	386 (7.9)	.001	0.08	161 (6.3)	163 (6.4)	.954	0.00
Healthcare utilization during 6‐month baseline, *n* (%)								
Emergency incidence	458 (15.9)	1119 (22.9)	<.001	0.18	720 (28.2)	723 (28.4)	.950	0.00
Endocrine outpatient incidence	286 (9.9)	400 (8.2)	.010	0.06	346 (13.6)	352 (13.8)	.839	0.01
Inpatient incidence	189 (6.6)	617 (12.6)	<.001	0.21	321 (12.6)	315 (12.4)	.832	0.01

Abbreviations: ACE, angiotensin‐converting enzyme; BMI, body mass index; DPP‐4, dipeptidyl peptidase‐4; Gla‐100, insulin glargine 100 units/mL; Gla‐300, insulin glargine 300 units/mL; GLP‐1 RA, glucagon‐like peptide‐1 receptor agonist; HbA1c, glycated haemoglobin A1c; HCP, healthcare provider; IDet, insulin detemir; N/A, not applicable; OAD, oral anti‐diabetic; RAI, rapid‐acting insulin; SD, standard deviation; SGLT2, sodium glucose co‐transporter 2; SMD, standard mean difference; US, United States.

† Last value during 6‐month baseline.

In the matched cohorts (Table 1), most patients were White, the mean age was 60.5 years, and 47.6% were male. Mean (SD) baseline HbA1c was 9.05% (1.87%) and 9.09% (1.93%) in the Gla‐300 and Gla‐100/IDet switcher cohorts, respectively. Similar proportions of patients in the two cohorts had HbA1c <7%, 7% to <8%, 8% to <9%, and ≥9%. Approximately 24–25% of patients in both switcher cohorts had recorded a hypoglycaemic event during the 12‐month baseline period.

### 3.2 HbA1c

**FIGURE 2:**
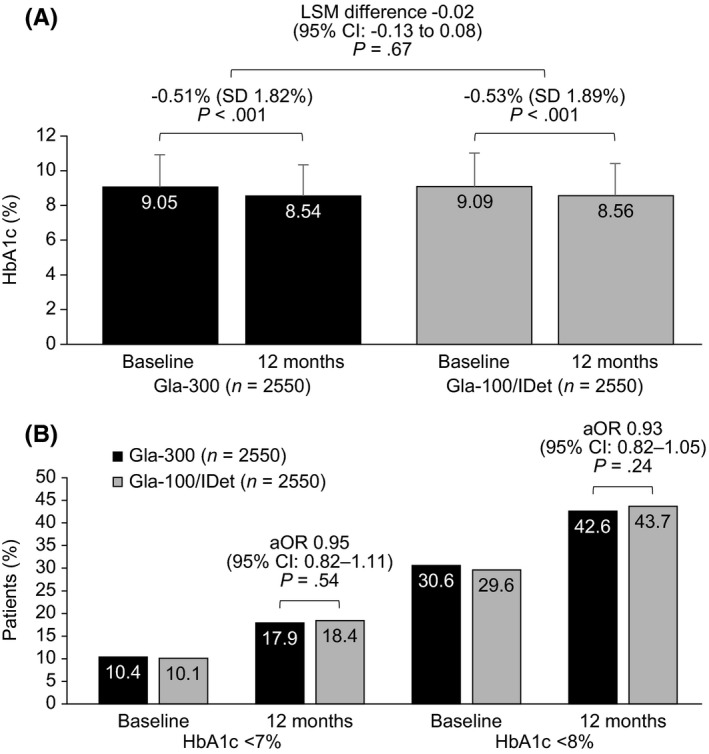
HbA1c outcomes at 12 months for patients with T2D switching to Gla‐300 or Gla‐100/IDet. (A) Reduction in HbA1c from baseline; (B) HbA1c goal attainment. Abbreviations: aOR, adjusted odds ratio; CI, confidence interval; Gla‐100, insulin glargine 100 units/mL; Gla‐300, insulin glargine 300 units/mL; HbA1c, glycated haemoglobin A1c; IDet, insulin detemir; LSM, least‐square mean; SD, standard deviation; T2D, type 2 diabetes

During the 12‐month follow‐up period, HbA1c decreased significantly from baseline in both cohorts (Figure 2A). The mean (SD) reductions in HbA1c were similar in the Gla‐300 and Gla‐100/IDet switcher cohorts: −0.51% (1.82%) and −0.53% (1.89%), respectively; LSM difference –0.02; 95% CI: –0.13 to 0.08; *p* = .67). Similar proportions of patients reached HbA1c <7% (17.9% vs. 18.4%, respectively; *p* = .54) and HbA1c <8% (42.6% vs. 43.7%, respectively; *p* = .24) (Figure 2B).

### 3.3 Hypoglycaemia

**FIGURE 3:**
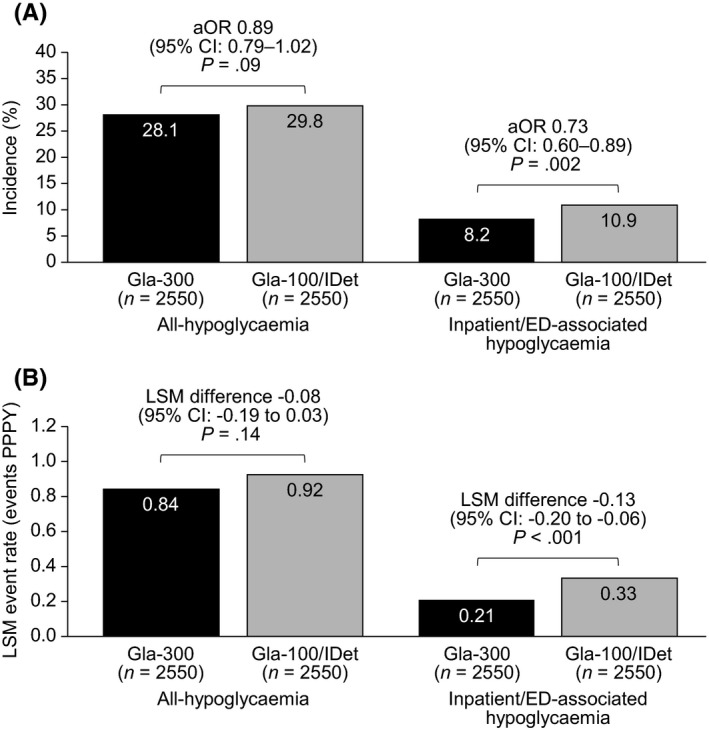
Hypoglycaemia outcomes at 12 months in patients with T2D switching to Gla‐300 or Gla‐100/IDet. (A) Incidences for all‐hypoglycaemia and inpatient/ED‐associated hypoglycaemia and odds ratios adjusted for baseline hypoglycaemia; (B) Adjusted event rates for all‐hypoglycaemia and inpatient/ED‐associated hypoglycaemia. Abbreviations: aOR, adjusted odds ratio; CI, confidence interval; ED, emergency department; Gla‐100, insulin glargine 100 units/mL; Gla‐300, insulin glargine 300 units/mL; IDet, insulin detemir; LSM, least‐square mean; PPPY, per patient per year; T2D, type 2 diabetes

During the 12‐month follow‐up period, patients in the Gla‐300 and Gla‐100/IDet switcher cohorts had comparable all‐hypoglycaemia incidence (28.1% vs. 29.8%, respectively), also after controlling for baseline all‐hypoglycaemia incidence and event rates (aOR: 0.89, 95% CI: 0.79–1.02; *p* = .09) (Figure 3A). They also experienced a comparable number of all‐hypoglycaemia events (adjusted mean events 0.84 vs. 0.92 PPPY, respectively; LSM difference –0.08; 95% CI: –0.19 to 0.03; *p* = .14) (Figure 3B).

Patients in the Gla‐300 switcher cohort had a significantly lower risk of experiencing hypoglycaemia associated with an inpatient/ED contact than patients in the Gla‐100/IDet cohort (8.2% vs. 10.9%, respectively); also after adjusting for baseline inpatient/ED‐associated hypoglycaemia (aOR: 0.73, 95% CI: 0.60–0.89; *p* = .002) (Figure 3A). Additionally, patients in the Gla‐300 switcher cohort experienced significantly fewer inpatient/ED‐associated hypoglycaemic events than patients in the Gla‐100/IDet cohort (0.21 vs. 0.33 events PPPY, respectively; LSM difference –0.13; 95% CI: –0.20 to –0.06; *p *< .001) (Figure 3B).

### 3.4 Subgroup analyses

The largest subgroup was subgroup 1 (uncontrolled HbA1c) with 3565 patients; the smallest was subgroup 7 (sulphonylurea use) with 1254 patients (Table 2). At 12‐month follow‐up, all subgroups showed HbA1c reductions from baseline, with no significant differences between Gla‐300 and Gla‐100/IDet switchers across the hypoglycaemia‐risk subgroups (Table 2). Gla‐300 switchers had a significantly lower all‐hypoglycaemia incidence than Gla‐100/IDet switchers in subgroups 3 (renal impairment), 4 (recent hypoglycaemia) and 6 (age ≥65 years), and a significantly lower all‐hypoglycaemia event rate in subgroups 3 (renal impairment) and 6 (age ≥65 years) (Table 2). Gla‐300 switchers had significantly lower inpatient/ED‐associated hypoglycaemia incidences than Gla‐100/IDet switchers in subgroups 1 (uncontrolled HbA1c), 2 (prandial insulin), 4 (recent hypoglycaemia), and 7 (sulphonylurea use) (Table 2). The inpatient/ED‐associated hypoglycaemia event rates were significantly lower in Gla‐300 switchers than in Gla‐100/IDet switchers in all seven subgroups (Table 2).

**TABLE 2:** High‐hypoglycaemia‐risk subgroup analyses for HbA1c and hypoglycaemia outcomes in Gla‐300 switchers versus Gla‐100/IDet switchers at 12 months

Subgroup	Patient, *n* (Gla−300; Gla−100/IDet)	Baseline differences^†^	HbA1c reduction, % (*P*‐value) LSM difference (95% CI)	All‐hypoglycaemia incidence, % (*P*‐value)	All‐hypoglycaemia event rate, PPPY (*P*‐value) LSM difference (95% CI)	Inpatient/ED‐associated hypoglycaemia incidence, % (*P*‐value)	Inpatient/ED‐associated hypoglycaemia event rate, PPPY (*P*‐value) LSM (95% CI)
Total high‐risk population (*n* = 5100)	2500; 2500	None	−0.51 vs. −0.53 (*p* = .670) LSM: −0.02 (−0.13 to 0.08)	28.1 vs. 29.8 (*p* = .089)	0.84 vs. 0.93 (*p* = .136) LSM: –0.08 (−0.19 to 0.03)	8.2 vs. 10.9 (*p* = .002)	0.21 vs. 0.33 (*p* <.001) LSM: –0.13 (−0.20 to −0.06)
Subgroup 1 Uncontrolled HbA1c (*n* = 3565)	1770; 1795	None	−0.88 vs. −0.91 (*p* = .991) LSM: −0.03 (−0.16 to 0.10)	28.8 vs. 29.0 (*p* = .923)	0.89 vs. 0.95 (*p* = .393) LSM: −0.06 (−0.20 to 0.08)	8.1 vs. 10.9 (*p* = .008)	0.21 vs. 0.36 (*p* = .001) LSM: −0.16 (−0.25 to −0.07)
Subgroup 2 Prandial insulin (*n* = 2662)	1344; 1318	Region	−0.44 vs. −0.53 (*p* = .265) LSM: −0.08 (−0.21 to 0.06)	30.4 vs. 34.5 (*p* = .053)	0.79 vs. 0.93 (*p* = .110) LSM: −0.14 (−0.31 to 0.03)	10.6 vs. 13.8 (*p* = .029)	0.24 vs. 0.42 (*p* = .002) LSM: −0.19 (−0.30 to −0.07)
Subgroup 3 Moderate‐to‐severe renal impairment (*n* = 1700)	861; 839	DPP‐4	−0.53 vs. −0.55 (*p* = .775) LSM: −0.03 (−0.21 to 0.15)	33.3 vs. 39.7 (*p* = .006)	0.98 vs. 1.29 (*p* = .006) LSM: −0.30 (−0.52 to −0.09)	13.5 vs. 16.7 (*p* = .079)	0.34 vs. 0.53 (*p* = .022) LSM: −0.18 (−0.34 to −0.03)
Subgroup 4 Recent hypoglycaemia (*n* = 2824)	1464; 1360	DPP‐4	−0.42 vs. –0.43 (*p* = .808) LSM: –0.02 (–0.15 to 0.12)	34.7 vs. 38.5 (*p* = .038)	1.14 vs. 1.22 (*p* = .338) LSM: –0.08 (–0.26 to 0.09)	10.7 vs. 14.9 (*p* = .004)	0.27 vs. 0.44 (*p* = .004) LSM: –0.17 (–0.28 to –0.06)
Subgroup 5 ASCVD (*n* = 1814)	908; 906	Baseline HbA1c	–0.56 vs. –0.38 (*p* = .316) LSM: 0.07 (–0.07 to 0.22)	36.9 vs. 38.7 (*p* = .405)	1.08 vs. 1.27 (*p* = .067) LSM: –0.19 (–0.40 to 0.01)	14.3 vs. 16.8 (*p* = .116)	0.37 vs. 0.51 (*p* = .036) LSM: –0.15 (–0.29 to –0.01)
Subgroup 6 Age ≥65 years (*n* = 2013)	1023; 990	Baseline BIA	–0.46 vs. –0.45 (*p* = .954) LSM: –0.00 (–0.15 to 0.14)	28.5 vs. 32.7 (*p* = .038)	0.82 vs. 1.00 (*p* = .037) LSM: –0.18 (–0.35 to –0.01)	9.6 vs. 12.2 (*p* = .098)	0.25 vs. 0.41 (*p* = .009) LSM: –0.15 (–0.27 to –0.04)
Subgroup 7 Sulphonylurea use (*n* = 1254)	615; 639	Metformin, HRU; hypoglycaemia events and endocrinology outpatient during 6‐month baseline	–0.33 vs. –0.52 (*p* = .084) LSM: –0.17 (–0.37 to 0.02)	25.0 vs. 24.6 (*p* = .876)	0.77 vs. 0.89 (*p* = .158) LSM: –0.13 (–0.30 to 0.05)	5.5 vs. 8.9 (*p* = .017)	0.06 vs. 0.22 (*p* = .001) LSM: –0.15 (–0.24 to –0.06)

Abbreviations: ASCVD, atherosclerotic cardiovascular disease; BIA, basal insulin analogue; CI, confidence interval; ED, emergency department; Gla‐100, insulin glargine 100 units/mL; Gla‐300, insulin glargine 300 units/mL; HbA1c, glycated haemoglobin A1c; HRU, healthcare resource utilization; IDet, insulin detemir; LSM, least‐square mean; PPPY, per patient per year.

† All *P*‐values are controlled for baseline differences.

### 3.5 Sensitivity analyses

The HbA1c sensitivity analysis results (ie, during 3‐ to 6‐ and 6‐ to 9‐month follow‐up) were very similar to the main results (ie, during 9‐ to 12‐month follow‐up) (Supporting Information Text S1).

When restricting hypoglycaemic events to those identified only by ICD‐9‐CM/ICD‐10‐CM codes, there were approximately half as many hypoglycaemia episodes, and the significant differences in inpatient/ED‐associated hypoglycaemia incidence and event rate in favour of Gla‐300 were lost (Supporting Information Text S2). Further, patients in the Gla‐300 switcher cohort experienced significantly more all‐hypoglycaemic events than those in the Gla‐100/IDet switcher cohort (*p* = .022).

## 4 DISCUSSION

In this large, retrospective real‐world study of EMR data of patients with T2D deemed at high risk of hypoglycaemia and treated with a BIA, switching to a different BIA – either second‐generation (Gla‐300) or first‐generation (Gla‐100 or IDet) – was associated with comparable glycaemic improvement and all‐hypoglycaemia incidence and event rates during 12 months of follow‐up, but significantly lower hypoglycaemia incidence and event rates associated with inpatient/ED contact.

In the current study, Gla‐300 switchers had significantly lower event rates associated with inpatient/ED visits than Gla‐100/IDet switchers in all seven risk subgroups. A reduction in event rates for hypoglycaemia associated with an inpatient stay or ED visit with Gla‐300 has also been found in the LIGHTNING study,^15^ which applied analytic approaches to EMR data to model and predict outcomes in patients with T2D. Patients at high risk of hypoglycaemia were predicted to have a non‐significantly lower risk of these hypoglycaemia events when switching to Gla‐300 versus Gla‐100 (0.21 vs. 0.26 per person‐year [PPY]) and a significantly lower risk versus IDet (0.21 vs. 0.30 PPY; *p* <.05). However, it should be noted that the LIGHTNING study did not specifically include patients with uncontrolled HbA1c, ASCVD or sulphonylurea use in their definition of a high‐hypoglycaemia‐risk group. Furthermore, the LIGHTNING study also used a definition to specifically capture severe hypoglycaemias, including inpatient/ED, ICD‐9‐CM/ICD‐10‐CM code relating to hypoglycaemic coma, plasma glucose <54 mg/dL, intramuscular glucagon administration, and ‘severe’ mentioned in the EMR.^15^ The LIGHTNING study reported significantly lower severe hypoglycaemia event rates for Gla‐300 versus IDet (but not Gla‐100) among patients with renal impairment, prandial insulin and aged ≥65 years, but not recent hypoglycaemia (using similar definitions to the current study).^15^ In DELIVER High Risk, patients aged ≥65 years who switched to Gla‐300 versus Gla‐100/IDet had significantly lower all‐hypoglycaemia incidence and event rates, and hypoglycaemia event rates associated with inpatient/ED visits. In DELIVER 3, which comprised only T2D patients aged ≥65 years,^20^ Gla‐300 switchers also had significantly lower hypoglycaemia incidences and event rates (all and inpatient/ED‐related) compared with Gla‐100/IDet switchers.

In the current study, three additional higher hypoglycaemia‐risk populations were explored, compared with the LIGHTNING study^15^ – uncontrolled HbA1c, ASCVD, and sulphonylurea users. The sulphonylurea user group generally had the lowest incidence and event rates (for all‐hypoglycaemia and inpatient/ED‐related hypoglycaemia) for either treatment group relative to the other risk subgroups, whereas the ASCVD subgroup generally ranked as the highest risk group in terms of incidence and event rates. Hypoglycaemia is thought to be associated with increased cardiovascular risk, potentially due to oxidative stress, inflammation, the development of atherosclerosis, etc.^31^, ^32^ It is also known that cardiovascular (CV) events increase the risk of severe hypoglycaemia events (SHEs) as well as the risk of CV events after SHEs, validating the bidirectional relationship between CV events and SHEs in patients with high comorbidity scores.^33^ Therefore, it may be that patients with ASCVD in the current study have a complex cycle of hypoglycaemic episodes with worsening ASCVD or increased frailty.

When examining the effect of Gla‐300 on HbA1c levels among patients at high risk of hypoglycaemia, mean HbA1c reductions from baseline among Gla‐300 switchers varied somewhat across the seven risk groups, from –0.33% (sulphonylurea use) to –0.88% (uncontrolled HbA1c), but there were no significant differences between Gla‐300 switchers and Gla‐100/IDet switchers in each subgroup. Similar results were found in high‐hypoglycaemia‐risk subgroup analyses in DELIVER D+,^19^ in which HbA1c reductions in the Gla‐300 arm varied from –0.54% (age ≥65 years) to –0.98% (uncontrolled HbA1c) (ASCVD and sulphonylurea use were not studied), with no significant differences between the Gla‐300 and insulin degludec arms. The mean overall HbA1c reduction in the Gla‐300 arm in the current study was –0.51% over 12 months, which was very similar to that found in general T2D adults in DELIVER D+ ^19^ and DELIVER 2 ^17^ (–0.63% and –0.51%, respectively) over the shorter period of 6 months. Further, attainment of HbA1c <7% and <8% was similar in the current study and in DELIVER D+ ^19^ and DELIVER 2,^17^ albeit over different follow‐up periods, indicating that Gla‐300 can be used effectively in high‐hypoglycaemia‐risk patients. In the current study, attainment of HbA1c <7% and <8% increased considerably from baseline to follow‐up (approximately 10% to 18% and 30% to 43%, respectively, in both arms) implying that despite having a higher hypoglycaemia risk, patients can still make important progress towards achieving glycaemic targets.

In the present study, 12‐month follow‐up data show that, despite higher rates of hypoglycaemia in this high‐risk population, patients on Gla‐300 generally had numerically lower incidences and event rates for all‐hypoglycaemia, and mainly significantly lower incidences and event rates for hypoglycaemia associated with inpatient/ED contacts, while at the same time reducing HbA1c compared with baseline, allowing more patients to achieve HbA1c goals.

### 4.1 Strengths and limitations

To our knowledge, this study is the first to provide real‐world insights into the characteristics of high‐hypoglycaemia‐risk patients and their clinical response to Gla‐300 vs Gla‐100/IDet over a 12‐month follow‐up period. Such patients are often excluded from randomized controlled trials, so these results provide valuable information for clinicians, healthcare‐delivery systems, patients and payers. However, the results of DELIVER High Risk should be interpreted with some caution because of its retrospective design and relatively short follow‐up (12 months). Further, diagnoses were based on ICD‐9‐CM/ICD‐10‐CM codes, but as EMR data may not link the actual diagnosis name, this could have resulted in some misclassification. Hypoglycaemias may have been underreported in the study (ie, there were no self‐monitoring‐blood‐glucose or continuous‐blood‐glucose‐monitoring data), and while events captured in EMR data and those in association with healthcare resource use may have been of clinical significance, the nature of those associations, in terms of cause and effect, cannot be easily determined with such records.

The use of propensity score‐matching ensured that the populations were well balanced on observed characteristics. However, as switching treatment regimens can be a complex decision (with both clinical and socioeconomic considerations) and EMR data may not reveal the reason why patients switched BIAs, selection bias may not be completely excluded even after propensity score‐matching. Also, patients who switched to Gla‐100/IDet were matched to those who switched to Gla‐300, potentially limiting the generalizability of the results. Data extracted from the database were mainly from patients from the Northwest and Southern states of the United States, and thus may not be representative of the US national landscape. Patients included in the study were required to have 12 months of follow‐up data so may not represent more recent users of second‐generation BIAs; therefore, their demographics and clinical characteristics may be different from BIA‐experienced patients in general.

EMRs only capture medication prescription, not dispensing or consumption; consequently, prescription information may not reflect actual drug usage in real life. Dosage data were missing in a high percentage of the EMRs; dose information and titration intensity/timing could not, therefore, be addressed in this study. Further, glycaemic goals for patients at increased risk of hypoglycaemia should be individualized.^34^ However, as it was not possible to explore individualized targets, all patients were measured against common HbA1c targets of <7% and <8%.

### 4.2 Conclusions

In conclusion, in patients with T2D at high risk of hypoglycaemia and treated with a BIA, switching to Gla‐300 versus a first‐generation BIA (Gla‐100 or IDet) resulted in similar HbA1c reductions and glycaemic goal attainment, but with a significantly lower risk of hypoglycaemia associated with inpatient/ED contacts (overall and in most risk subgroups) during the 12 months after switching.

## CONFLICT OF INTERESTS

S.D.S. has served on the advisory panels for, and received research support from, Boehringer Ingelheim, Novo Nordisk and Sanofi. N.F. has received funding for research, travel and consulting from Sanofi‐Aventis, AstraZeneca, Ipsen, PTC Therapeutics, Tesaro, Takeda, Akcea, MSD, Allergan. R.A.G. is an employee of Accenture, which was under contract with Sanofi. J. Wu., C.J.N., and J. We. are employees of, and stockholders in, Sanofi. T.S.B. has received research support from Abbott Diabetes, Abbott Rapid Diagnostics, Biolinq, Capillary Biomedical, Dexcom, Eli Lilly, Kowa, Lexicon, Livongo, Medtronic, Novo Nordisk, REMD, Sanofi, Sanvita, Senseonics, Viacyte, vTv Therapeutics, Zealand Pharma; consultant's honoraria from Abbott, Lifescan, Medtronic, Novo, Sanofi; and speaker's honoraria from BD, Medtronic, Sanofi.

## AUTHOR CONTRIBUTIONS

**S** **ean D Sullivan:** Conceptualization (equal); Formal analysis (equal); Methodology (equal); Writing‐review & editing (lead). **Nick Freemantle:** Conceptualization (equal); Formal analysis (equal); Methodology (equal); Writing‐review & editing (equal). **Rishab A Gupta:** Conceptualization (equal); Data curation (equal); Formal analysis (equal); Investigation (equal); Methodology (equal); Writing‐review & editing (equal). **Jasmanda Wu:** Conceptualization (equal); Formal analysis (equal); Methodology (equal); Writing‐review & editing (equal). **Charlie J Nicholls:** Conceptualization (equal); Formal analysis (equal); Methodology (equal); Writing‐review & editing (equal). **Jukka Westerbacka:** Conceptualization (equal); Formal analysis (equal); Methodology (equal); Writing‐review & editing (equal). **T. S. Bailey:** Conceptualization (equal); Formal analysis (equal); Methodology (equal); Writing‐review & editing (equal).

## HUMAN STUDIES AND SUBJECTS

The IBM Explorys data services provide aggregated, de‐identified data in accordance with the requirements of HIPAA. Given the observational and retrospective nature of this study, individual consent was not required after ensuring for anonymization of data.

## Supporting information

Supplementary Material

## Data Availability

Aggregate data are provided in the manuscript. Scripts used for pulling data from IBM Explorys platform are available on request from Rishab.a.gupta@accenture.com. Restrictions apply to the availability of the source patient‐level data, which were used under licence for this study.
