# Supplementary material for: Clinical outcomes in high‐hypoglycaemia‐risk patients with type 2 diabetes switching to insulin glargine 300 U/mL versus a first‐generation basal insulin analogue in the United States : Results from the DELIVER High Risk real‐world study
Source: Endocrinol Diabetes Metab. 2021 Nov 22;5(1):e00306. doi: 10.1002/edm2.306 (PMC8754248; doi:10.1002/edm2.306)
Supplement: Supplementary file 1 — Supplementary Material [file EDM2-5-e00306-s001.docx]

**Supporting Information**

# **Clinical outcomes in high-hypoglycaemia-risk patients with type 2 diabetes switching to insulin glargine 300 U/mL versus a first-generation basal insulin analogue in the US: Results from the DELIVER High Risk real-world study**

**Running title:** Clinical outcomes in high-hypoglycaemia-risk patients (DELIVER High Risk)

Sean D. Sullivan, Nick Freemantle, Rishab A. Gupta, Jasmanda Wu, Charlie J. Nicholls, Jukka Westerbacka, Timothy S. Bailey

**TABLE S1** Codes and conditions used to identify patients with T1D, T2D and hypoglycaemia

**TABLE S2** International Classification of Diseases codes used to identify comorbidities

**TEXT S1** HbA1c sensitivity analysis results

**TEXT S2** Hypoglycaemia sensitivity analysis results

# **TABLE S1** Codes and conditions used to identify patients with T1D, T2D and hypoglycaemia

| **T1D** | **T2D** | **T1D^†^ identified by one of the following:** | **Hypoglycaemia^‡^ identified by one of the following:** |
| --- | --- | --- | --- |
| ICD-9-CM codes: 250.X1 and 250.X3  ICD-10-CM codes: E10.XX | ICD-9-CM codes: 250.X0 and 250.X2  ICD-10-CM codes: E11.65, E11.00, E11.01, E11.36, E11.618, E11.621, E11.630, E11.29, E11.69, E11.311, E11.40, E11.51, E11.620, E11.622, E11.649, E11.8, E11.319, E11.39, E11.9, E11.641, E11.21, E11.628, E11.638 | Ratio of T1D to T2D ICD diagnoses >0.5 and one of the following:   - A prescription for glucagon - No record of anti-diabetic prescriptions other than metformin - Drug record of metformin and diagnosis of polycystic ovarian syndrome and no record of anti-diabetic prescription other than metformin; *OR*   C-peptide laboratory test <0.17 nmol/L  (<0.51 ng/mL) | ICD-9-CM codes: 251.0, 251.1, 251.2, 270.3  ICD-9-CM codes: 250.8X without concomitant 259.8, 272.7, 681.XX, 682.XX, 686.9X, 707.1-707.9, 709.3, 730.0-730.2, 731.8  ICD-10-CM codes: E0864, E08641, E08649, E0964, E09641, E09649, E1064, E10641, E10649, E1164, E11641, E11649, E1364, E13641, E13649, E15, E160, E161, E162  Blood glucose ≤70 mg/dL (3.9 mmol/L) |

†T1D was identified using the algorithm developed by Klompas et al. *Diabetes Care* 2013;36:914–921.

‡Hypoglycaemia was identified using the algorithm developed by Ginde et al. *BMC Endocr Disord* 2008;8:4.

Abbreviations: ICD-9-CM/ICD-10-CM, International Classification of Diseases, Ninth or Tenth Revisions, Clinical Modification; T1D, type 1 diabetes; T2D, type 2 diabetes

# **TABLE S2** International Classification of Diseases (ICD) codes used to identify comorbidities

| **Comorbidity** | **ICD-9-CM codes** | **ICD-10-CM codes** |
| --- | --- | --- |
| Hypertension | 401.X-405.XX, 437.2 | I10-I15.X, I67.4 |
| Hyperlipidaemia | 272.0 - 272.4 | E78.0-E78.5 |
| Obesity | 278.00, 278.01, 278.03, V85.3X, V85.4X | E66.0X, E66.1, E66.2, E66.8, E66.9, Z68.3XX, Z68.4XX |
| Neuropathy | 249.6, 250.6 | E08.40-E08.43, E09.40-E09.43, E10.40-E10.43, E11.40-E11.43, E13.40-E13.43 |
| Depression | 296.2, 296.3, 296.5, 300.4, 309.x, 311 | F20.4, F31.3-F31.5, F32.x, F33.x, F34.1, F41.2, F43.2 |
| Retinopathy | 249.5, 362.0X | E08.31-E08.35, E09.31-E09.35, E10.31-E10.35, E11.31-E11.35, E13.31-E13.35 |
| Nephropathy | 249.4, 250.4 | E08.21, E09.21, E10.21, E11.21, E13.21 |

Abbreviations: ICD-9-CM/ICD-10-CM, International Classification, Ninth or Tenth Revisions, Clinical Modification.

**TEXT S1** HbA1c sensitivity analysis results

Among patients with an HbA1c measurement during 3- to 6-month follow-up, mean baseline HbA1c was 8.99% in the Gla-300 switcher cohort (1668 patients) versus 8.99% in the Gla-100/IDet switcher cohort (1623 patients). HbA1c levels decreased significantly to 8.44% and 8.54%, respectively, in the two cohorts (*P* < .0001 for both). Mean (SD) HbA1c reductions were comparable in both cohorts: −0.55% (1.69%) versus –0.45% (1.71%), respectively; LSM difference 0.10 (95% CI: –0.02 to 0.22; *P* = .10. Patients in the Gla-300 and Gla-100/IDet cohorts also showed comparable HbA1c goal attainment, with similar proportions of patients achieving HbA1c <7.0% (15.4% vs. 16.8%, respectively; aOR 0.85; 95% CI: 0.70–1.04; *P* = .11) and <8.0% (44.7% vs. 42.8%, respectively; aOR 1.02; 95% CI: 0.87 to 1.19; *P* = .81).

Among patients with an HbA1c measurement during 6- to 9-month follow-up, mean baseline HbA1c was 8.99% in the Gla-300 switcher cohort (1500 patients) and 9.09% in the Gla-100/IDet switcher cohort (1385 patients). HbA1c levels significantly decreased to 8.51% and 8.75% in the two cohorts, respectively (*P* < .0001 for both). HbA1c reductions were comparable in both cohorts: –0.48% (1.68%) versus –0.52% (1.72%), respectively; LSM difference –0.04 (95% CI: –0.16 to 0.09; *P* = .57). Patients in the Gla-300 and Gla-100/IDet switcher cohorts showed comparable HbA1c goal attainment, with similar proportions of patients achieving HbA1c <7.0% (15.4% vs. 16.2%, respectively; aOR 0.90; 95% CI: 0.72–1.11; *P* = .32) and <8.0% (42.4% vs. 41.6%, respectively; aOR 0.98; 95% CI: 0.83–1.15; *P* = .82).

**TEXT S2** Hypoglycaemia sensitivity analysis results

Restricting hypoglycaemic events to those identified only by ICD-9-CM/ICD-10-CM codes and controlling for baseline hypoglycaemia incidence and event rate, patients in the Gla-300 switcher and Gla-100/IDet switcher cohorts had comparable all-hypoglycaemia risk during the 12-month follow-up period (19.1% vs. 17.0%, respectively; aOR 1.15; 95% CI: 0.99–1.34; *P* = .08). All-hypoglycaemia event rate was 0.56 PPY in the Gla-300 switcher and 0.46 PPY in the Gla-100/IDet switcher cohort during the 12-month follow-up period (LSM difference 0.09; 95% CI: 0.01–0.17; *P* = .022).

For inpatient/ED-associated hypoglycaemic events, a similar proportion of patients in the Gla-300 and Gla-100/IDet switcher cohorts experienced events (3.7% and 4.0%, respectively; aOR 0.94; 95% CI: 0.70–1.26; *P* = .67). Patients in the Gla-300 and Gla-100/IDet switcher cohorts experienced comparable inpatient/ED-associated hypoglycaemic events during the 12-month follow-up (LSM events 0.05 vs. 0.07 PPPY, respectively; LSM difference –0.01; 95% CI: –0.04 to 0.01; *P* = .20).
